# Technological advancements in surgical laparoscopy considering artificial intelligence: a survey among surgeons in Germany

**DOI:** 10.1007/s00423-023-03134-6

**Published:** 2023-10-16

**Authors:** Sebastian Lünse, Eric L. Wisotzky, Sophie Beckmann, Christoph Paasch, Richard Hunger, René Mantke

**Affiliations:** 1 Department of General and Visceral Surgery, Brandenburg Medical School, University Hospital Brandenburg/Havel, Hochstrasse 29, 14770 Brandenburg, Germany; 2 Vision and Imaging Technologies, Fraunhofer Heinrich-Hertz-Institut HHI, Einsteinufer 37, 10587 Berlin, Germany; 3 Department of Computer Science, Humboldt-Universität Zu Berlin, Unter Den Linden 6, 10117 Berlin, Germany; 4 Faculty of Health Science Brandenburg, Brandenburg Medical School, University Hospital Brandenburg/Havel, 14770 Brandenburg, Germany

**Keywords:** Laparoscopic surgery, Artificial intelligence, Survey

## Abstract

**Purpose:**

The integration of artificial intelligence (AI) into surgical laparoscopy has shown promising results in recent years. This survey aims to investigate the inconveniences of current conventional laparoscopy and to evaluate the attitudes and desires of surgeons in Germany towards new AI-based laparoscopic systems.

**Methods:**

A 12-item web-based questionnaire was distributed to 38 German university hospitals as well as to a Germany-wide voluntary hospital association (CLINOTEL) consisting of 66 hospitals between July and November 2022.

**Results:**

A total of 202 questionnaires were completed. The majority of respondents (88.1%) stated that they needed one assistant during laparoscopy and rated the assistants’ skillfulness as “very important” (39.6%) or “important” (49.5%). The most uncomfortable aspects of conventional laparoscopy were inappropriate camera movement (73.8%) and lens condensation (73.3%). Selected features that should be included in a new laparoscopic system were simple and intuitive maneuverability (81.2%), automatic de-fogging (80.7%), and self-cleaning of camera (77.2%). Furthermore, AI-based features were improvement of camera positioning (71.3%), visualization of anatomical landmarks (67.3%), image stabilization (66.8%), and tissue damage protection (59.4%). The reason for purchasing an AI-based system was to improve patient safety (86.1%); the reasonable price was €50.000–100.000 (34.2%), and it was expected to replace the existing assistants’ workflow up to 25% (41.6%).

**Conclusion:**

Simple and intuitive maneuverability with improved and image-stabilized camera guidance in combination with a lens cleaning system as well as AI-based augmentation of anatomical landmarks and tissue damage protection seem to be significant requirements for the further development of laparoscopic systems.

**Supplementary Information:**

The online version contains supplementary material available at 10.1007/s00423-023-03134-6.

## Introduction

The surgical laparoscopy marked a fundamental step in the evolution of medicine and the technical development of the procedure progressed consistently since its first application on humans more than hundred years ago.

The primary focus of current advancements is to enhance image quality and achieve greater realism through the implementation of 3D technology [1]. Additionally, there is ongoing progress in the field of robotic-assisted surgery, as well as the exploration of new surgical approaches, such as natural orifice transluminal endoscopic surgery (NOTES) and the single-port technique [2–4].

Artificial intelligence (AI) has been rapidly developing in recent years, and the latest form of computer-based automation that self-analyzes a large amount of data is encompassing nearly all aspects of daily life, represented by self-driving cars. Global interest in AI applications, particularly in surgery, has increased significantly in the recent years [5]. Continuous improvements in computing technology, the accessibility of big data, and the rise of machine learning have paved the way for the increasing use of AI in surgery [6]. Laparoscopic systems that are based on AI have the potential to improve real-time performance by analyzing the large amount of visual data generated during laparoscopy [7]. Visualization of intraoperative anatomy with the aid of augmented or virtual reality [8, 9], AI-assisted surgical training [10], or control of surgical instruments by recognizing gestures [11] are some examples of current AI applications. Nevertheless, the lack of registration accuracy within real-world conditions has hindered the transfer into clinical practice [12].

Therefore, we conducted this web-based survey among German surgeons in academic and non-academic hospitals to investigate the experiences of physicians performing minimally invasive surgery, the limitations of conventional laparoscopic surgical systems, as well as the attitudes and desires towards AI-based laparoscopic surgical systems. Thus, the present survey may help to highlight the weaknesses of conventional laparoscopy and further advance the development of AI-based laparoscopic systems to enable future implementation into surgical routine.

## Materials and methods

### Survey

The prospective survey was conducted anonymously in accordance with the General Data Protection Regulation (GDPR) using the “SoSci” web-application, a commercially available e-survey platform [13]. An invitation to participate in the survey was sent by e-mail to practicing surgeons of general, visceral, thoracic, oncological, and vascular surgery from all 38 German university hospitals as well as from the CLINOTEL hospital association (voluntary association of 38 public and 28 non-profit hospitals distributed throughout Germany) between July 1st and November 30th, 2022. The questionnaire was modified from a previous survey by Park et al. [14] and included 12 items (see supplemental material Table 1). The questionnaire was in German and single or multiple answers were possible, depending on the question type. As this study only used anonymized data among medical professionals, a waiver was granted according to the local ethics committee (E-01–20230705).

### Data analysis

The survey data were analyzed by using descriptive statistics. The position of the surveyed surgeons was considered as ordinal variable in the analysis, as were variables with multicategory response formats (e.g., working hospital, work experience). Associations between these variables were analyzed using the Mantel–Haenszel linear-by-linear chi-square test. For the evaluation of binary variables (agreement vs. disagreement) and an ordinal variable, the Cochran–Armitage trend test was used. The analysis was performed using R (the R Foundation, version 4.2.2). All tests were two-sided and a *p* value < 0.05 was set as the significance threshold.

## Results

### Demographics and surgical experience

From the 2686 invited surgeons, 215 responded (8%) of which 202 were completed and analyzed. The majority of respondents (*n* = 166, 82.2%) worked at university hospitals. More than half of the surgeons performed more than five laparoscopic procedures per month (*n* = 148, 73.3%). In terms of the assistance during laparoscopic surgery, the majority (*n* = 178, 88.1%) preferred one assistant. With regard to the importance of the skillfulness of assistants for successful laparoscopic surgery, the majority of respondents indicated “very important” (*n* = 80, 39.6%) or “important” (*n* = 100, 49.5%). Data is also shown in the supplemental material Table 2.

### Limitations of conventional laparoscopic systems

**Fig. 1:**
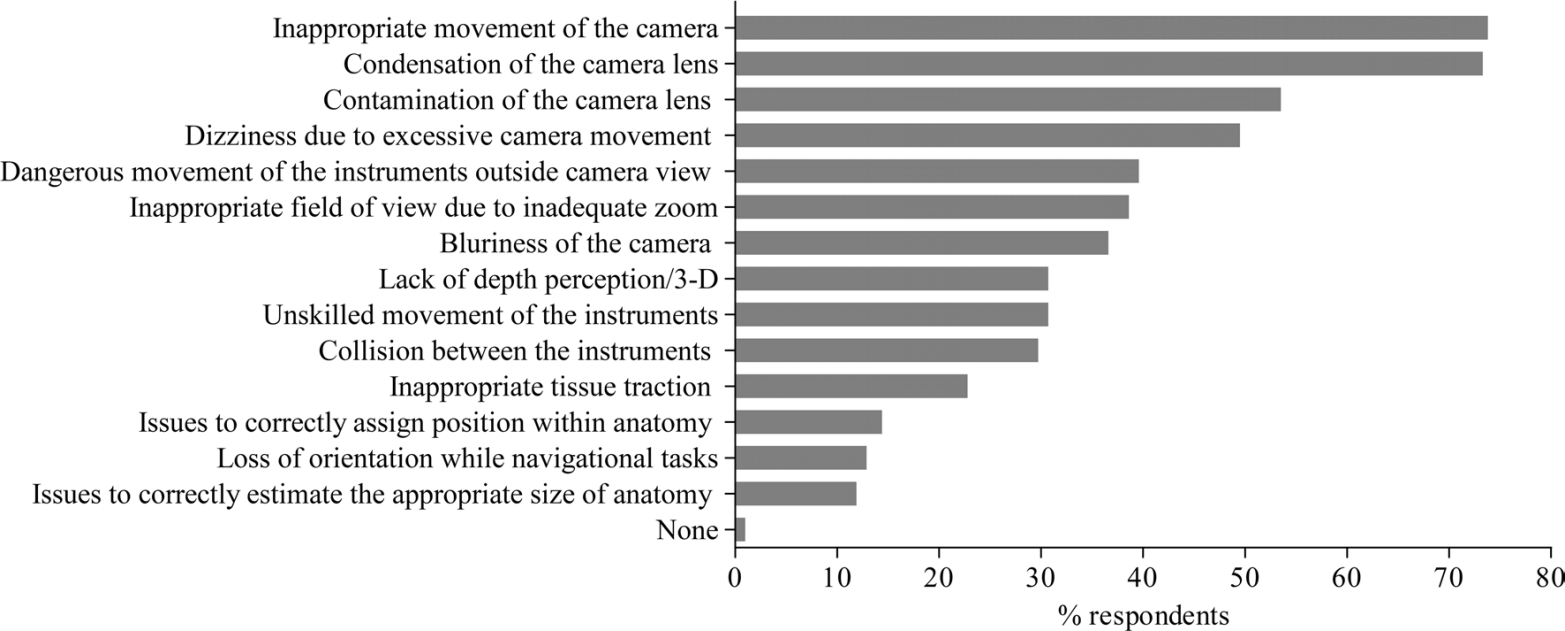
Perceived inconvenience of camera and instrument assistance with conventional laparoscopic systems. Responses were selected from a list of choices; multiple selections were possible

The surgeons’ responses to the questions concerning the limitations of conventional laparoscopic surgical systems regarding camera and instrument assistance are shown in Fig. 1. In terms of camera assistance, almost three-quarters of the respondents were unsatisfied with an inappropriate movement of camera (*n* = 149, 73.8%) and with the condensation of camera lens (*n* = 148, 73.3%). Regarding instrument assistance, the most limitations of conventional laparoscopic surgical systems are dangerous movement of the instruments outside camera view (*n* = 80, 39.6%), unskilled movement of the instruments (*n* = 62, 30.7%), and collision between the instruments (*n* = 60, 29.7%). Related to differences between surgical training levels, head physicians were statistically significantly dissatisfied with inappropriate movement of camera (*n* = 23, 92%, *p* < 0.001) and instruments (*n* = 10, 40%, *p* = 0.048). Senior physicians indicated inappropriate tissue traction (*n* = 26, 32.9%, *p* = 0.009), and resident physicians reported issues to correctly estimate the appropriate size of anatomy (*n* = 13, 18.6%, *p* = 0.025), loss of orientation while navigational tasks (*n* = 15, 21.4%, *p* = 0.004), and collision between the instruments (*n* = 30, 42.9%, *p* = 0.008). Data is also shown in the supplemental material Table 3.

### Attitudes and desires towards AI-based laparoscopic surgical systems

**Fig. 2:**
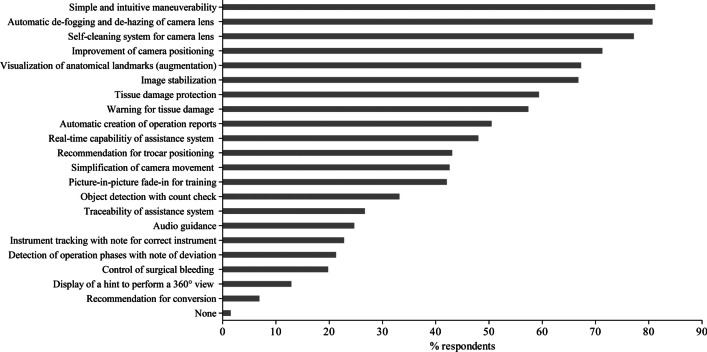
Features that should be included in an AI-based laparoscopic surgical system reported by participants; multiple selections were possible

**Fig. 3:**
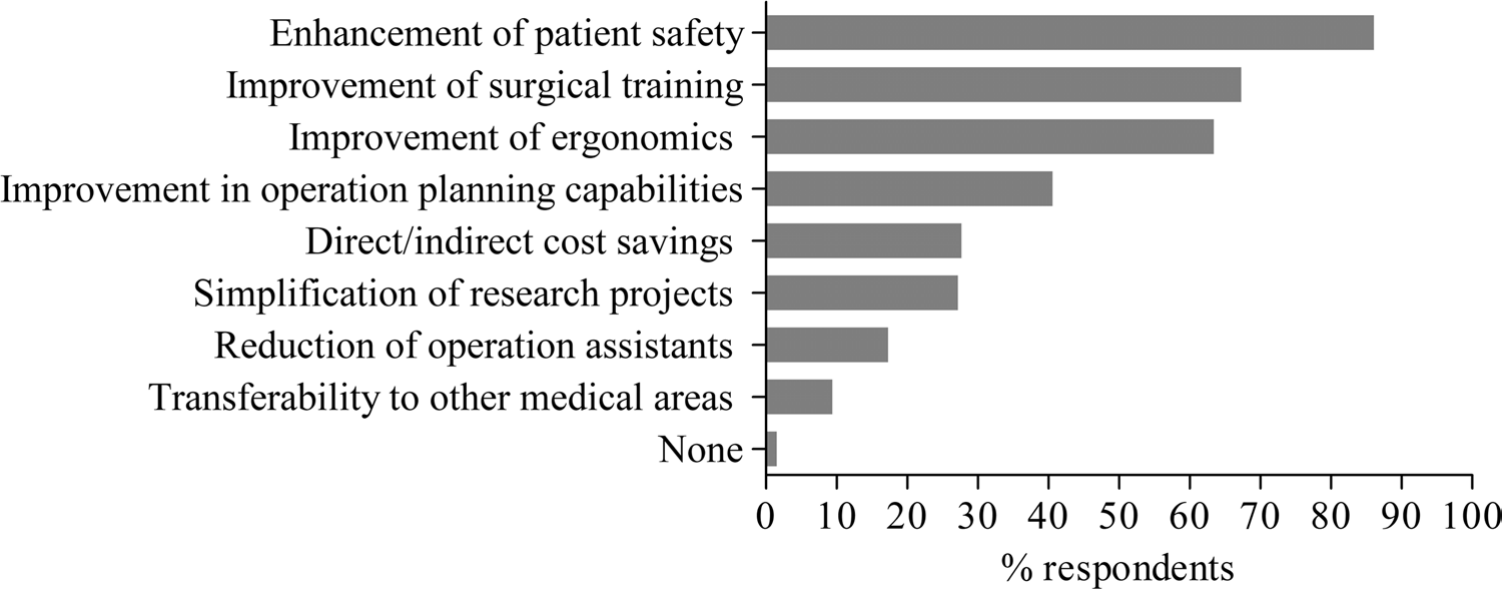
Reasons for purchasing an AI-based laparoscopic surgical system selected by the participants; multiple selections were possible

**Fig. 4:**
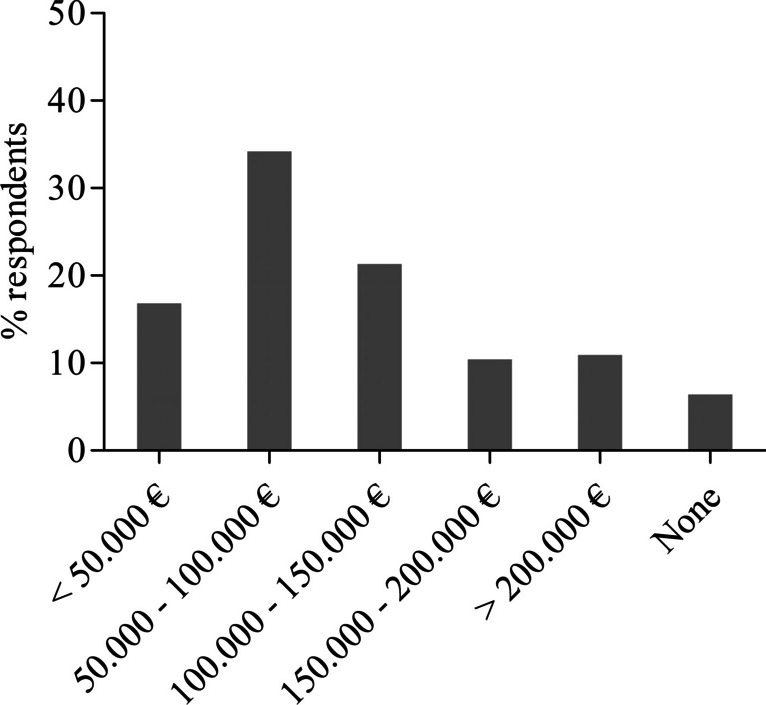
Price for an AI-based laparoscopic surgical system that participants considered reasonable

**Fig. 5:**
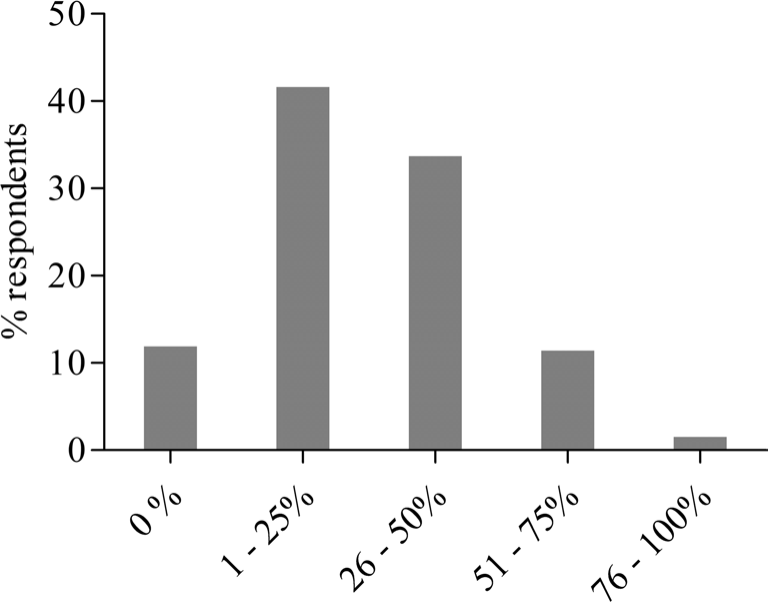
Participants’ expectations for the possibility of replacing the existing workflow of surgical assistants by an artificial intelligence-based laparoscopic surgical system

The responses to questions on the surgeons’ desires related to particular functions that should be included in an AI-based surgical assistant system to overcome the limitations of current conventional laparoscopic systems are shown in Fig. 2. More than three-fourths of the respondents preferred simple and intuitive maneuverability (*n* = 164, 81.2%), automatic de-fogging and de-hazing of camera lens (*n* = 163, 80.7%), and a self-cleaning system for camera lens (*n* = 156, 77.2%). Furthermore, more than half of the respondents expressed their desire for an improvement of camera positioning (*n* = 144, 71.3%), visualization of anatomical landmarks (*n* = 136, 67.3%), image stabilization (*n* = 135, 66.8%), a tissue damage protection system (*n* = 120, 59.4%) as well as warning for tissue damage (*n* = 116, 57.4%), and automatic creation of operation reports (*n* = 102, 50.5%), respectively. Related to differences between surgical training levels, head physicians were statistically significantly interested in the real-time capability of assistance system (*n* = 16, 64%, *p* = 0.007), which means that an event is responded to within a precisely defined period of time that is as short as possible, and resident physicians in picture-in-picture fade-in for training (*n* = 35, 50%, *p* = 0.035), respectively. When asked about the reasons for buying an artificial intelligence-based laparoscopic surgical system, the clear majority of respondents stated enhancement of patient safety (*n* = 174, 86.1%) followed by improvement of surgical training (*n* = 136, 67.3%) and ergonomics (*n* = 128, 63.4%), as shown in Fig. 3. Head physicians were statistically significantly interested in an improvement of ergonomics (*n* = 20, 80%, *p* = 0.050) and resident physician in an improvement of surgical training (*n* = 56, 80%, *p* = 0.002). Regarding the reasonable price, 34.2% (*n* = 69) of the respondents were willing to pay €50,000–100,000 (Fig. 4). On the question of what percentage of the assistant’s work could be replaced by AI in the future, the majority of the respondents (*n* = 84, 41.6%) expected a substitute between 1 and 25%, as shown in Fig. 5. Data is also shown in the supplemental material Tables 4–6.

## Discussion

One of the most outstanding achievements in the history of medicine is the integration of laparoscopy into surgical practice, and minimally invasive surgery has evolved at an incredible pace over the past two decades [15]. Artificial intelligence is a key technology of the twenty-first century and it will change people’s lives in the coming decades [16]. Surgery is an area of medicine that benefits greatly from technological developments and particularly surgical laparoscopy has increasingly come into the focus of AI research in recent years [17]. We conducted this survey to give insights in expectations and demands of surgeons performing minimally invasive surgery in Germany to support the further development of current laparoscopy as well as new AI-based systems.

The majority of surgeons participating in the survey preferred one assistant during laparoscopic surgery and highly valued that assistant’s skills in handling the laparoscopic camera and instruments. A survey by Park et al. with 508 Korean participants revealed very similar findings [14]. In this context, the experience of the assistant seems to be closely related to operation time and rate of adverse effects [18].

Regarding the limitations of current laparoscopic systems, most of the interviewed surgeons felt uncomfortable with vision restrictions due to inappropriate camera movement as well as condensation or contamination of the camera lens. Interestingly, there were some statistically significant differences between surgical training levels. Experienced surgeons, such as head and senior physicians, were particularly bothered by insufficient camera and instrument movements and tissue tractions, while resident physicians had problems with the estimation of anatomical sizes and intraoperative orientation as well as collision of laparoscopic instruments. These results confirm the laparoscopic learning curve with different problems according to the level of surgical training. However, artificial intelligence has enormous potential to significantly improve laparoscopic training in the next few years through the use of virtual and/or augmented reality [19, 20].

The most frequently stated wishes regarding essential functions of a future AI-based laparoscopic surgical system were simple and intuitive maneuverability followed by automatic de-fogging and de-hazing of camera lens as well as a self-cleaning system for the lens. This confirms the results concerning inconvenience with conventional laparoscopic systems, where an inappropriate camera movement as well as condensation and contamination of the camera lens were most frequently stated. New AI-based features, e.g., improvement of camera positioning, visualization of anatomical landmarks, image stabilization, tissue damage protection, and warning system as well as automatic creation of operation reports were indicated as further desirable functions of an AI-based laparoscopic system by more than 50% of the respondents. A main reason for this subordinate weighting of AI-features is probably insufficient knowledge of new AI technologies among the surgeons surveyed. In an online survey of 296 German surgeons by Wilhelm et al., only about 20% of the respondents stated that they had a good or very good self-assessed level of knowledge regarding artificial intelligence and decision-support systems [21].

However, these findings underline the highly important role of assistance in surgical laparoscopy. In the near future, a symbiosis between artificial intelligence and already established robotic surgery, such as the DaVinci system, could play a pioneering role in minimally invasive surgery [22, 23].

Enhancement of patient safety was the main reason for the vast majority of survey participants to purchase an AI-based assistance system for laparoscopies. The in-hospital mortality after visceral surgery is not uncommon in Germany, with a frequency of nearly 2%. However, the use of artificial intelligence has great potential to significantly enhance surgical safety in a sustainable manner [24, 25]. An area of active research is the use of AI to recognize critical procedural steps as well as anatomy in intraoperative laparoscopic videos. For example, AI-based systems can already identify the steps of laparoscopic sleeve gastrectomy with an accuracy of 82% [26] and the achievement of critical view of safety in laparoscopic cholecystectomy, yielding an accuracy of 71.4% [27].

Other frequently given reasons for participants to buy an AI-based system are improvements in surgical training as well as ergonomics. Comparing the different levels of training, head physicians significantly favored improving ergonomics during laparoscopy, which may be primarily due to the increased number of surgical procedures and longer operation times. In contrast, resident physicians were significantly interested in improving surgical training, as expected. However, AI has improved surgical education in recent year by significantly increasing its accuracy and capability [28]. The increasing use of surgical simulators in modern medicine can be enhanced by AI technology through personalized feedback to the user, identification of patient anatomy, 3D visualization devices, and augmented reality [29–33]. Consistently, a study by Yang et al. showed an improvement in basic surgical skills by an AI-assisted hands-on training [10].

When asked about the possible purchase price of an AI-based laparoscopic surgical system, more than half of the respondents showed willingness to spend between 50,000 and 150,000 euros. However, current studies do not allow conclusions to be drawn regarding the cost-effectiveness of AI solutions in terms of clinical, technical, and economic viability [34].

The majority of the surveyed surgical community in the present study expects a replacement of the current workflow of surgical assistance during laparoscopy by AI-based systems up to 25% in the future. This fundamental open-mindedness of German surgeons towards technical developments through the use of artificial intelligence has already been demonstrated by previous studies [21, 35, 36]. Due to the worldwide rapidly growing role, a Delphi consensus defined the common term “digital surgery” in the year 2022 as “the use of technology for the enhancement of preoperative planning, surgical performance, therapeutic support, or training, to improve outcomes and reduce harm” [37].

The present study has some limitations, including the small sample size of 202 participants, which is not representative of all regions in Germany. Additionally, response bias cannot be excluded due to voluntary participation. Moreover, the survey population is more homogeneous compared to previous publications [36], as it was conducted exclusively in Germany. Therefore, the results should be complemented by international surveys. With a response rate of 8%, this survey is about in the lower third compared to the response rates of comparable studies by Park et al. with 3.5%, Pecqueux et al. with 37.5%, or Cobianchi et al. with approximately 70% [14, 35, 36]. Another bias is the varying laparoscopic experience of the surgeons surveyed. While at 73.3%, almost three-quarters of the respondents perform more than five laparoscopies per month, about a quarter with less than 5 monthly procedures have significantly less practical experience. This affects the response behavior, as each surgical training level has its own requirements and the difficulty and complexity of laparoscopic procedures increases with professional experience. A selection bias is generated by the fact that 82.2% of the respondents in this study are employed at university hospitals. The proportion of surgeons from non-university hospitals cannot therefore be considered representative, and no further conclusions can be drawn from this environment. With regard to the university task of research, the interest and acceptance of AI as well as the possibility of practical AI implementations are probably higher in university hospitals than in non-academic institutions.

Nevertheless, due to the homogeneous group of participants as well as the high number of participants, the present study reflects a good opinion on the technical further development of surgical laparoscopy including artificial intelligence among selected surgeons in Germany.

## Conclusion

In conclusion, the present survey of surgeons in Germany confirmed the high value of the assistance in surgical laparoscopy, whereby inappropriate camera movement and viewing restrictions of the camera lens are highlighted limitations of current conventional laparoscopic systems. Simple and intuitive maneuverability with improved and image-stabilized camera guidance in combination with a lens cleaning system as well as augmentation of anatomical landmarks and tissue damage protection seem to be significant requirements for the further development of laparoscopic systems.

### Supplementary Information

Below is the link to the electronic supplementary material.

Supplementary file1 (DOCX 87 KB)

Supplementary file2 (DOCX 87 KB)

Supplementary file3 (DOCX 83 KB)

Supplementary file4 (DOCX 106 KB)

Supplementary file5 (DOCX 82 KB)

Supplementary file6 (DOCX 51 KB)

## Data Availability

The study data will be available from the corresponding author for review upon a reasonable request.
