# Supplementary material for: Technological advancements in surgical laparoscopy considering artificial intelligence: a survey among surgeons in Germany
Source: Langenbecks Arch Surg. 2023 Oct 16;408(1):405. doi: 10.1007/s00423-023-03134-6 (PMC10579134; doi:10.1007/s00423-023-03134-6)
Supplement: Supplementary file 1 — Supplementary file1 (DOCX 87 KB) [file 423_2023_3134_MOESM1_ESM.docx]

**Supplementary Table 1.** Questionnaire details.

| Variable and question number | Question and multiple choice answer options |
| --- | --- |
| **Experience** |  |
| 1 | What type of hospital do you work at? |
| 2 | What is your level of training? |
| 3 | What is your work experience? |
| 4 | How many laparoscopic interventions do you perform monthly? |
| 5 | How many assistants do you need during laparoscopic surgery? |
| 6 | How important is the skillfulness of your assistant during laparoscopic surgery? |
| **Limitation** |  |
| 7 | What are the technical difficulties of the conventional laparoscopic surgical system from your point of view? (multiple choice)   - Inappropriate movement of the camera - Dizziness due to excessive camera movement - Inappropriate field of view due to inadequate zoom - Lack of depth perception/3-D - Issues to correctly estimate the appropriate size of anatomy - Issues to correctly assign position within anatomy - Condensation of the camera lens - Contamination of the camera lens - Bluriness of the camera - Unskilled movement of the instruments - Inappropriate tissue traction - Dangerous movement of the instruments outside camera view - Loss of orientation while navigational tasks - Collision between the instruments - None |
| **Attitude and desire** | |
| 8 | Where is the use of an AI-based surgical assistant system especially useful? (multiple choice)   - Detection of operation phases with note of deviation - Visualization of anatomical landmarks (augmentation) - Instrument tracking with note for correct instrument - Object detection with count check - Control of surgical bleeding - Improvement of camera positioning - Picture-in-picture fade-in for training - Warning for tissue damage - Display of a hint to perform a 360° view - Recommendation for trocar positioning - Recommendation for conversion - Automatic creation of operation reports - None |
| 9 | What particular functions do you want to be included in an AI-based surgical assistant system? (multiple choice)   - Simple and intuitive maneuverability - Image stabilization - Tissue damage protection - Audio guidance - Self-cleaning system for camera lens - Automatic de-fogging and de-hazing of camera lens - Simplification of camera movement - Real-time capability of assistance system - Traceability of assistance system - None |
| 10 | Why would you want to buy an AI-based surgical assistant system?  (multiple choice)   - Enhancement of patient safety - Improvement in operation planning capabilities - Improvement of ergonomics - Reduction of operation assistants - Improvement of surgical training - Transferability to other medical areas - Direct/indirect cost savings - Simplification of research projects - None |
| 11 | How much would you be willing to pay for an AI-based surgical assistant system? |
| 12 | What percentage of your assistant’s work could be replaced by an AI-based surgical assistant system in the future? |
