# Supplementary material for: Technological advancements in surgical laparoscopy considering artificial intelligence: a survey among surgeons in Germany
Source: Langenbecks Arch Surg. 2023 Oct 16;408(1):405. doi: 10.1007/s00423-023-03134-6 (PMC10579134; doi:10.1007/s00423-023-03134-6)
Supplement: Supplementary file 2 — Supplementary file2 (DOCX 87 KB) [file 423_2023_3134_MOESM2_ESM.docx]

**Supplementary Table 2.** Demographic data.

| Answers | Total  (N=202), n (%) | Head physician  (N=25), n (%) | Senior physician  (N=79), n (%) | Consultant  (N=28), n (%) | Resident physician (N=70), n (%) | *P* value |
| --- | --- | --- | --- | --- | --- | --- |
| **Working hospital** |  |  |  |  |  | **0.002** |
| University | 166 (82.2%) | 9 (36%) | 69 (87.3%) | 27 (96.4%) | 61 (87.1%) |  |
| Maximum care | 8 (4%) | 5 (20%) | 1 (1.3%) | 1 (3.6%) | 1 (1.4%) |  |
| Advanced care | 20 (9.9%) | 8 (32%) | 8 (10.1%) | 0 (0%) | 4 (5.7%) |  |
| General | 8 (4%) | 3 (12%) | 1 (1.3%) | 0 (0%) | 4 (5.7%) |  |
| **Work experience (years)** |  |  |  |  |  | **< 0.001** |
| ≤ 5 | 47 (23.3%) | 0 (0%) | 0 (0%) | 0 (0%) | 47 (67.1%) |  |
| 6-10 | 59 (29.3%) | 0 (0%) | 14 (17.7%) | 22 (78.6%) | 23 (32.9%) |  |
| 11-20 | 53 (26.2%) | 3 (12%) | 46 (58.2%) | 4 (14.3%) | 0 (0%) |  |
| 21-30 | 34 (16.8%) | 16 (64%) | 16 (20.2%) | 2 (7.1%) | 0 (0%) |  |
| ≥ 31 | 9 (4.5%) | 6 (24%) | 3 (3.8%) | 0 (0%) | 0 (0%) |  |
| **Laparoscopic surgeries per month** |  |  |  |  |  | **< 0.001** |
| 0 | 12 (5.9%) | 0 (0%) | 1 (1.3%) | 1 (3.6%) | 10 (14.3%) |  |
| 1-5 | 42 (20.8%) | 1 (4%) | 5 (6.3%) | 6 (21.4%) | 30 (42.9%) |  |
| 6-10 | 52 (25.7%) | 5 (20%) | 19 (24.1%) | 8 (28.6%) | 20 (28.6%) |  |
| 11-20 | 60 (29.7%) | 8 (32%) | 34 (43%) | 11 (39.3%) | 7 (10%) |  |
| 21-30 | 18 (8.9%) | 3 (12%) | 13 (16.5%) | 1 (3.6%) | 1 (1.4%) |  |
| ≥ 31 | 18 (8.9%) | 8 (32%) | 7 (8.9%) | 1 (3.6%) | 2 (2.9%) |  |
| **Assistants during laparoscopic surgery** |  |  |  |  |  | 0.409 |
| 0 | 2 (1%) | 0 (0%) | 0 (0%) | 0 (0%) | 2 (2.9%) |  |
| 1 | 178 (88.1%) | 22 (88%) | 69 (87.3%) | 27 (96.4%) | 60 (85.7%) |  |
| 2 | 22 (10.9%) | 3 (12%) | 10 (12.7%) | 1 (3.6%) | 8 (11.4%) |  |
| ≥ 3 | 0 (0%) | 0 (0%) | 0 (0%) | 0 (0%) | 0 (0%) |  |
| **Importance of the assistants’ skillfulness** |  |  |  |  |  | 0.102 |
| Very important (1) | 80 (39.6%) | 8 (32%) | 27 (34.2%) | 8 (28.6%) | 37 (52.9%) |  |
| Important (2) | 100 (49.5%) | 13 (52%) | 44 (55.7%) | 18 (64.3%) | 25 (35.7%) |  |
| Neutral (3) | 15 (7.4%) | 2 (8%) | 6 (7.6%) | 2 (7.1%) | 5 (7.1%) |  |
| Rather not important (4) | 5 (2.5%) | 2 (8%) | 1 (1.3%) | 0 (0%) | 2 (2.9%) |  |
| Unimportant (5) | 2 (1%) | 0 (0%) | 1 (1.3%) | 0 (0%) | 1 (1.4%) |  |
