# Supplementary material for: Technological advancements in surgical laparoscopy considering artificial intelligence: a survey among surgeons in Germany
Source: Langenbecks Arch Surg. 2023 Oct 16;408(1):405. doi: 10.1007/s00423-023-03134-6 (PMC10579134; doi:10.1007/s00423-023-03134-6)
Supplement: Supplementary file 3 — Supplementary file3 (DOCX 83 KB) [file 423_2023_3134_MOESM3_ESM.docx]

**Supplementary Table 3.** Limitations of conventional laparoscopic surgical systems.

| Answers | Total (N=202), n (%) | Head physician  (N=25),  n (%) | Senior physician  (N=79),  n (%) | Consultant (N=28),  n (%) | Resident physician (N=70), n (%) | *P* value |
| --- | --- | --- | --- | --- | --- | --- |
| **Camera assistance** |  |  |  |  |  |  |
| Inappropriate movement of the camera | 149 (73.8%) | 23 (92%) | 65 (82.3%) | 20 (71.4%) | 41 (58.6%) | **< 0.001** |
| Dizziness due to excessive camera movement | 100 (49.5%) | 13 (52%) | 42 (53.2%) | 14 (50%) | 31 (44.3%) | 0.306 |
| Inappropriate field of view due to inadequate zoom | 78 (38.6%) | 12 (48%) | 32 (40.5%) | 10 (35.7%) | 24 (34.3%) | 0.214 |
| Lack of depth perception/3-D | 62 (30.7%) | 6 (24%) | 19 (24%) | 12 (42.9%) | 25 (35.7%) | 0.085 |
| Issues to correctly estimate the appropriate size of anatomy | 24 (11.9%) | 2 (8%) | 5 (6.3%) | 4 (14.3%) | 13 (18.6%) | **0.025** |
| Issues to correctly assign position within anatomy | 29 (14.4%) | 3 (12%) | 13 (16.5%) | 4 (14.3%) | 9 (12.9%) | 0.774 |
| Condensation of the camera lens | 148 (73.3%) | 20 (80%) | 56 (70.9%) | 22 (78.6%) | 50 (71.4%) | 0.681 |
| Contamination of the camera lens | 108 (53.5%) | 13 (52%) | 40 (50.6%) | 18 (64.3%) | 37 (52.9%) | 0.738 |
| Bluriness of the camera | 74 (36.6%) | 8 (32%) | 35 (44.3%) | 11 (39.3%) | 20 (28.6%) | 0.201 |
| **Instrument assistance** |  |  |  |  |  |  |
| Unskilled movement of the instruments | 62 (30.7%) | 10 (40%) | 28 (35.4%) | 8 (28.6%) | 16 (22.9%) | **0.048** |
| Inappropriate tissue traction | 46 (22.8%) | 6 (24%) | 26 (32.9%) | 6 (21.4%) | 8 (11.4%) | **0.009** |
| Dangerous movement of the instruments outside camera view | 80 (39.6%) | 8 (32%) | 30 (38%) | 8 (28.6%) | 34 (48.6%) | 0.127 |
| Loss of orientation while navigational tasks | 26 (12.9%) | 1 (4%) | 6 (7.6%) | 4 (14.3%) | 15 (21.4%) | **0.004** |
| Collision between the instruments | 60 (29.7%) | 5 (20%) | 19 (24%) | 6 (21.4%) | 30 (42.9%) | **0.008** |
| **None** | 2 (1%) | 0 (0%) | 1 (1.3%) | 1 (3.6%) | 0 (0%) | 0.783 |
