# Supplementary material for: Technological advancements in surgical laparoscopy considering artificial intelligence: a survey among surgeons in Germany
Source: Langenbecks Arch Surg. 2023 Oct 16;408(1):405. doi: 10.1007/s00423-023-03134-6 (PMC10579134; doi:10.1007/s00423-023-03134-6)
Supplement: Supplementary file 4 — Supplementary file4 (DOCX 106 KB) [file 423_2023_3134_MOESM4_ESM.docx]

**Supplementary Table 4.** Surgeons’ desires regarding functions that should be included in an artificial intelligence-based laparoscopic surgical system.

| Function | Total (N=202), n (%) | Head physician  (N=25), n (%) | Senior physician  (N=79), n (%) | Consultant (N=28), n (%) | Resident physician (N=70), n (%) | *P* value |
| --- | --- | --- | --- | --- | --- | --- |
| Simple and intuitive maneuverability | 164 (81.2%) | 23 (92%) | 63 (79.7%) | 24 (85.7%) | 54 (77.1%) | 0.233 |
| Image stabilization | 135 (66.8%) | 19 (76.0%) | 53 (67.1%) | 20 (71.4%) | 43 (61.4%) | 0.232 |
| Audio guidance | 50 (24.7%) | 9 (36.0%) | 22 (27.8%) | 4 (14.3%) | 15 (21.4%) | 0.114 |
| Tissue damage protection | 120 (59.4%) | 12 (48.0%) | 52 (65.8%) | 18 (64.3%) | 38 (54.3%) | 0.693 |
| Self-cleaning system for camera lens | 156 (77.2%) | 19 (76.0%) | 59 (74.7%) | 24 (85.7%) | 54 (77.1%) | 0.688 |
| Automatic de-fogging and de-hazing of camera lens | 163 (80.7%) | 20 (80.0%) | 61 (77.2%) | 22 (78.6%) | 60 (85.7%) | 0.271 |
| Simplification of camera movement | 86 (42.6%) | 14 (56%) | 30 (38.0%) | 8 (28.6%) | 34 (48.6%) | 0.882 |
| Real-time capability of assistance system | 97 (48%) | 16 (64%) | 43 (54.4%) | 12 (42.9%) | 26 (37.1%) | **0.007** |
| Traceability of assistance system | 54 (26.7%) | 8 (32%) | 17 (21.5%) | 8 (28.6%) | 21 (30.0%) | 0.576 |
| Detection of operation phases with note of deviation | 43 (21.3%) | 7 (28%) | 16 (20.3%) | 8 (28.6%) | 12 (17.1%) | 0.383 |
| Visualization of anatomical landmarks (augmentation) | 136 (67.3%) | 14 (56%) | 53 (67.1%) | 20 (71.4%) | 49 (70.0%) | 0.279 |
| Tracking of instruments with note for correct instrument | 46 (22.8%) | 6 (24%) | 12 (15.2%) | 9 (32.1%) | 19 (27.1%) | 0.186 |
| Object detection with count check | 67 (33.2%) | 10 (40%) | 25 (31.6%) | 10 (35.7%) | 22 (31.4%) | 0.632 |
| Control of surgical bleeding | 40 (19.8%) | 5 (20%) | 11 (13.9%) | 4 (14.3%) | 20 (28.6%) | 0.078 |
| Improvement of camera positioning | 144 (71.3%) | 20 (80%) | 56 (70.9%) | 18 (64.3%) | 50 (71.4%) | 0.567 |
| Picture-in-picture fade-in for training | 85 (42.1%) | 7 (28%) | 30 (38.0%) | 13 (46.4%) | 35 (50.0%) | **0.035** |
| Warning for tissue damage | 116 (57.4%) | 15 (60%) | 48 (60.8%) | 17 (60.7%) | 36 (51.4%) | 0.281 |
| Display of a hint to perform a 360° view | 26 (12.9%) | 5 (20%) | 7 (8.9%) | 4 (14.3%) | 10 (14.3%) | 0.907 |
| Recommendation for trocar positioning | 87 (43.1%) | 10 (40%) | 27 (34.2%) | 14 (50.0%) | 36 (51.4%) | 0.056 |
| Recommendation for conversion | 14 (6.9%) | 4 (16%) | 3 (3.8%) | 1 (3.6%) | 6 (8.6%) | 0.814 |
| Automatic creation of operation reports | 102 (50.5%) | 13 (52%) | 41 (51.9%) | 13 (46.4%) | 35 (50.0%) | 0.772 |
| **None** | 3 (1.5%) | 1 (4%) | 1 (1.3%) | 1 (3.6%) | 0 (0.0%) | 0.249 |
