# Supplementary material for: Technological advancements in surgical laparoscopy considering artificial intelligence: a survey among surgeons in Germany
Source: Langenbecks Arch Surg. 2023 Oct 16;408(1):405. doi: 10.1007/s00423-023-03134-6 (PMC10579134; doi:10.1007/s00423-023-03134-6)
Supplement: Supplementary file 5 — Supplementary file5 (DOCX 82 KB) [file 423_2023_3134_MOESM5_ESM.docx]

**Supplementary Table 5.** Factors and reasonable prices to buy an artificial intelligence-based laparoscopic surgical system.

| Answers | Total (N=202), n (%) | Head physician  (N=25), n (%) | Senior physician  (N=79), n (%) | Consultant (N=28), n (%) | Resident physician (N=70), n (%) | *P* value |
| --- | --- | --- | --- | --- | --- | --- |
| **Factors to buy the system** |  |  |  |  |  |  |
| Enhancement of patient safety | 174 (86.1%) | 23 (92%) | 67 (84.8%) | 22 (78.6%) | 63 (90%) | 0.830 |
| Improvement in operation planning capabilities | 82 (40.6%) | 10 (40%) | 34 (43%) | 11 (39.3%) | 27 (38.6%) | 0.683 |
| Improvement of ergonomics | 128 (63.4%) | 20 (80%) | 53 (67.1%) | 15 (53.6%) | 41 (58.6%) | **0.050** |
| Reduction of operation assistants | 35 (17.3%) | 2 (8%) | 19 (24%) | 6 (21.4%) | 8 (11.4%) | 0.407 |
| Improvement of surgical training | 136 (67.3%) | 14 (56%) | 46 (58.2%) | 20 (71.4%) | 56 (80%) | **0.002** |
| Transferability to other medical areas | 19 (9.4%) | 3 (12%) | 4 (5.1%) | 5 (17.9%) | 7 (10%) | 0.566 |
| Direct/indirect cost savings | 56 (27.7%) | 7 (28%) | 23 (29.1%) | 10 (35.7%) | 16 (22.9%) | 0.496 |
| Simplification of research projects | 55 (27.2%) | 5 (20%) | 24 (30.4%) | 8 (28.6%) | 18 (25.7%) | 0.992 |
| **None** | 3 (1.5%) | 1 (4%) | 1 (1.3%) | 1 (3.6%) | 0 (0%) | 0.249 |
|  |  |  |  |  |  |  |
| **Reasonable price (€)** |  |  |  |  |  | 0.594† |
| ≤ 50.000 | 34 (16.8%) | 3 (12%) | 15 (19%) | 6 (21.4%) | 10 (14.3%) |  |
| 50.000 – 100.000 | 69 (34.2%) | 8 (32%) | 29 (36.7%) | 12 (42.9%) | 20 (28.6%) |  |
| 100.000 – 150.000 | 43 (21.3%) | 2 (8%) | 17 (21.5%) | 6 (21.4%) | 18 (25.7%) |  |
| 150.000 – 200.000 | 21 (10.4%) | 5 (20%) | 5 (6.3%) | 2 (7.1%) | 9 (12.9%) |  |
| ≥ 200.000 | 22 (10.9%) | 3 (12%) | 9 (11.4%) | 1 (3.6%) | 9 (12.9%) |  |
| **None** | 13 (6.4%) | 4 (16%) | 4 (5.1%) | 1 (3.6%) | 4 (5.7%) |  |

Note. † the reported p-value is based on a test where the “none” category was discarded, if “none” is considered to be zero, the resulting p-value is 0.319
