# Supplementary material for: Technological advancements in surgical laparoscopy considering artificial intelligence: a survey among surgeons in Germany
Source: Langenbecks Arch Surg. 2023 Oct 16;408(1):405. doi: 10.1007/s00423-023-03134-6 (PMC10579134; doi:10.1007/s00423-023-03134-6)
Supplement: Supplementary file 6 — Supplementary file6 (DOCX 51 KB) [file 423_2023_3134_MOESM6_ESM.docx]

**Supplementary Table 6.** Expectations on the possibility of replacing the existing workflow of surgical assistants by an artificial intelligence-based laparoscopic surgical system.

| Answers | Total (N=202), n (%) | Head physician  (N=25), n (%) | Senior physician  (N=79), n (%) | Consultant (N=28), n (%) | Resident physician (N=70), n (%) | *P* value |
| --- | --- | --- | --- | --- | --- | --- |
| 0% | 24 (11.9%) | 4 (16%) | 12 (15.2%) | 2 (7.1%) | 6 (8.6%) | 0.775 |
| 1 – 25% | 84 (41.6%) | 12 (48%) | 24 (30.4%) | 16 (57.1%) | 32 (45.7%) |  |
| 26 – 50% | 68 (33.7%) | 7 (28%) | 28 (35.4%) | 9 (32.1%) | 24 (34.3%) |  |
| 51 – 75% | 23 (11.4%) | 2 (8%) | 14 (17.7%) | 1 (3.6%) | 6 (8.6%) |  |
| 76 – 100% | 3 (1.5%) | 0 (0%) | 1 (1.3%) | 0 (0%) | 2 (2.9%) |  |
